# A qualitative research framework for the design of user-centered displays of explanations for machine learning model predictions in healthcare

**DOI:** 10.1186/s12911-020-01276-x

**Published:** 2020-10-08

**Authors:** Amie J. Barda, Christopher M. Horvat, Harry Hochheiser

**Affiliations:** 1 Department of Biomedical Informatics, School of Medicine, University of Pittsburgh, 5607 Baum Boulevard, Pittsburgh, PA 15206 USA; 2 Children’s Hospital of Pittsburgh of UPMC, Pittsburgh, PA 15224 USA; 3 Department of Critical Care Medicine, University of Pittsburgh School of Medicine, Pittsburgh, PA 15213 USA; 4 Safar Center for Resuscitation Research, University of Pittsburgh, Pittsburgh, PA 15224 USA; 5 Brain Care Institute, Children’s Hospital of Pittsburgh of UPMC, Pittsburgh, PA 15261 USA; 6 Intelligent Systems Program, University of Pittsburgh, Pittsburgh, PA 15213 USA

**Keywords:** Machine learning, Explainable artificial intelligence, User-computer interface, Clinical decision support systems, In-hospital mortality, Pediatric intensive care units

## Abstract

**Background:**

There is an increasing interest in clinical prediction tools that can achieve high prediction accuracy and provide explanations of the factors leading to increased risk of adverse outcomes. However, approaches to explaining complex machine learning (ML) models are rarely informed by end-user needs and user evaluations of model interpretability are lacking in the healthcare domain. We used extended revisions of previously-published theoretical frameworks to propose a framework for the design of user-centered displays of explanations. This new framework served as the basis for qualitative inquiries and design review sessions with critical care nurses and physicians that informed the design of a user-centered explanation display for an ML-based prediction tool.

**Methods:**

We used our framework to propose explanation displays for predictions from a pediatric intensive care unit (PICU) in-hospital mortality risk model. Proposed displays were based on a model-agnostic, instance-level explanation approach based on feature influence, as determined by Shapley values. Focus group sessions solicited critical care provider feedback on the proposed displays, which were then revised accordingly.

**Results:**

The proposed displays were perceived as useful tools in assessing model predictions. However, specific explanation goals and information needs varied by clinical role and level of predictive modeling knowledge. Providers preferred explanation displays that required less information processing effort and could support the information needs of a variety of users. Providing supporting information to assist in interpretation was seen as critical for fostering provider understanding and acceptance of the predictions and explanations. The user-centered explanation display for the PICU in-hospital mortality risk model incorporated elements from the initial displays along with enhancements suggested by providers.

**Conclusions:**

We proposed a framework for the design of user-centered displays of explanations for ML models. We used the proposed framework to motivate the design of a user-centered display of an explanation for predictions from a PICU in-hospital mortality risk model. Positive feedback from focus group participants provides preliminary support for the use of model-agnostic, instance-level explanations of feature influence as an approach to understand ML model predictions in healthcare and advances the discussion on how to effectively communicate ML model information to healthcare providers.

## Background

Although numerous prior efforts have demonstrated the successful application of machine learning (ML) models to complex problems in medicine, there is a distinct absence of these models in practical applications [1–3]. Many discussions attribute this absence to a lack of model interpretability, commonly described as the ability of a human to understand factors contributing to a model’s behavior [4–6]. With increasing societal concerns and regulations on intelligent algorithms, [6–8] recognition of the importance of incorporating providers’ and domain knowledge in modeling processes, [1, 3, 6, 9, 10] and provider demand for model explanations, [2, 4–6] interpretability will be vital to the future success of ML models in healthcare.

The ML community has developed several approaches to explaining models and predictions. Although user goals, expertise, and time constraints play critical roles in determining what information an explanation must provide to meet user needs, [11–13] there is an apparent lack of end-user involvement in the design and evaluation of explanation approaches. The definition of what constitutes a “good” or “useful” explanation is often left to the judgment of novice and expert model developers, whose knowledge and backgrounds are generally not representative of end-user expertise [11]. Developers are often exclusively concerned with the statistical and modeling challenges of generating an explanation; the display of the explanation often receives less attention and is rarely informed by end-user needs or insights from the literature [11, 12, 14]. Current evaluation studies provide limited insight into how end-users interpret and utilize explanations designed by modeling experts, [5] particularly if accurate interpretation of those models requires some level of understanding of ML models. This may lead to a lack of usability and practical interpretability of these explanations for real end-users.

Researchers in the human computer interaction (HCI) and ML communities have proposed frameworks for and provided guidance on user-centered design for explainable ML and artificial intelligence (AI) [14–19]. This literature focuses mainly on *who* an explanation is provided to—the user of the system, and *why* the user requires an explanation—the specific goals the user is trying to accomplish [15, 17, 19]. While these are important elements in understanding the context of use of an explanation, little attention seems to be paid to *where* or *when* users require explanations. These questions relate to the environment in which a user is expected to use an explanation, which can impact how an explanation needs to be designed and evaluated. Environment is particularly important to consider when designing displays of explanation tools for ML models to be used in clinical decision support systems (CDSS), as research has shown that CDSS that interfere with existing workflows are unlikely to be used and accepted by clinicians [20].

Our goal was to use clinician perspectives to inform the design of a user-centered display of an explanation for an ML-based prediction tool. More specifically, we propose an explanation display design framework that considers the *entire* context of use of an explanation and can thus account for healthcare provider explanation needs when utilizing a predictive model in clinical practice. We demonstrate an application of our framework by using it to suggest possible explanation displays for a pediatric intensive care unit (PICU) in-hospital mortality risk model. Finally, we solicit pediatric critical care provider feedback on proposed displays to gain a better understanding of explanation needs, identify successful display elements, and inform the design of a user-centered display of an explanation for the PICU in-hospital mortality risk model.

### Framework for designing user-centered displays of explanation

Although previous conceptual frameworks and guidance for user-centered explainable ML and AI [14–18] have included the user perspective, key contextual and goal-related questions have not been discussed. To close this gap, we revise and extend existing frameworks—particularly those of Wang, et al. [15] and Ribera and Lapedriza [17]—to explicitly include contextual factors.

The framework by Wang et al. [15] relies on theories of human reasoning and explanation, and highlights specific elements of AI explanation that support these processes and mitigate errors. It promotes explanation design by linking specific AI explanation techniques and elements to the human cognitive patterns they can support (e.g., “what if” type explanations support counterfactual reasoning) and covers many concepts and techniques from the literature on model interpretability. Although useful for identifying explanation techniques to support specific reasoning goals and cognitive processes, this model does not account for how these factors may differ by specific types of users or how environmental constraints on the user may affect these processes. Ribera and Lapedriza [17] rely on theories that describe explanation as a social interaction and present a framework focused on understanding explainee (i.e., user) needs according to their background and relationship to the AI system. They define general user types and identify specific explanation goals (why), the content to include in an explanation (what), the type of explanation or explanation approach (how), and suitable evaluation approaches for each user type. Although the framework helps elucidate general design elements to support the explanation goals for each user type, it includes only a small portion of concepts and techniques from the model interpretability literature and it also does not consider how environment may affect explanation needs.

**Fig. 1:**
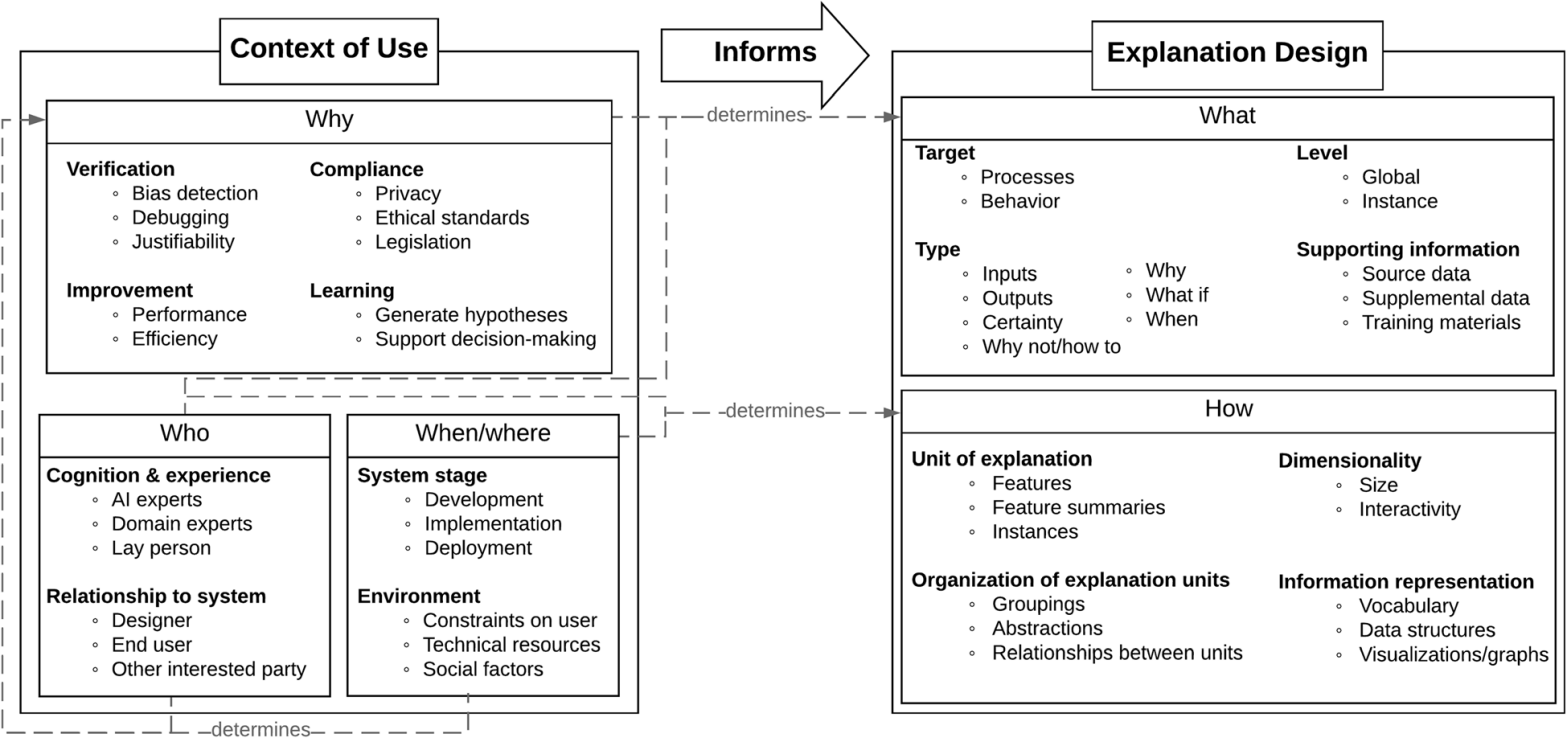
Proposed framework for designing user-centered displays of explanation

Our framework (Fig. 1) extends the *why*, *what*, *how* structure of explanation introduced by Ribera and Lapedriza [17] to include *when* and *where* an explanation will be used. Following Wang et al. [15]‘s approach, our suggestions for explanation content (*what*) and presentation (*how*) encompass many concepts from the model interpretability literature; however, rather than mapping specific reasoning processes to existing explanation techniques, we consider the broader picture of how context of use elements map to general concepts of explanation from the literature. More specifically, our framework suggests that answering target questions about the context of use (*who*, *why*, *when*, *where*) can help answer target questions about explanation design choices such as *what* information the explanation needs to contain (i.e., the content) and *how* that information needs to be provided (i.e., the presentation). Fig. 1 lists general factors that should be considered for each target question (e.g., cognition and experience for *who*) along with a few specific examples for each factor (e.g., AI expert). These factors are discussed along with supporting literature in the next few sections. As indicated by the grey dashed lines in Fig. 1, the target questions are not orthogonal and are often co-dependent in that the answers to one question can and will be determined by the answers to other target questions.

#### Who

Prior work has tried to create categories of users to define explanation needs, but, as discussed in Ras et al. [14], users often fall into multiple categories. We instead argue that users can generally be defined by two aspects: 1) user cognition and experience and 2) the user’s relationship to the system at the time the explanation is provided. Ribera and Lapedriza’s [17] classifications of AI experts, domain experts, and lay persons capture the main categories of user cognition that appear in the literature. Work by Ras et al. [14] provides sub-categorizations of users (engineer, developer, owner, end-user, data subject, stakeholder), capturing the various relationships a user may have with an AI system. In our model (Fig. 1), these sub-categorizations are generalized into the role of designer (engineer, developer), end user, and other stakeholders (owner, data subject, stakeholder). These dimensions overcome the problem of trying to create mutually exclusive user categories to define needs. A user may have several different relationships with a system over time, and thus their explanation needs may change with varying roles.

#### When/where

A broad classification of *when* and *where* an explanation is being used can be related to the stage of the system, which often defines a user’s relationship to the system (e.g., during system development the user relationship to the system is often that of designer). Explanations required during system development, implementation, and deployment will likely differ in design due to the different environmental settings associated with each system stage. *When/where* can be answered by considering the environment in which the explanation will be used and how the explanation needs to be designed in order to support use within that environment. Specifically, environment will dictate the constraints on the user (e.g., available time and cognitive capacity), the available technical resources, and social factors influencing the user’s perception of the system, which are all elements that may influence explanation needs.

#### Why

This target question depends on the answers to the *who* and *when/where* target questions, as the user, their relationship to the system, and the environment in which they will operate often affects why an explanation is sought. Although several prior efforts have identified various user needs and goals that drive the need for explanations, most of these can be captured in four broad categories defined by Samek et al. [21]: 1) verification—examining how decisions are made by the system to ensure it is operating as expected; 2) improvement—improving system performance, efficiency, and/or utility; 3) learning—extracting knowledge from the system; and 4) compliance—ensuring the system adheres to an established legal, moral or other societal standard. It should be noted that these are not mutually exclusive categories (e.g., explanations for verification are also often used to guide improvement activities). When users request explanations in the context of decision-making, they are generally requesting explanations for verification (e.g., support for a specific decision suggested by the system) and/or explanations for learning (e.g., knowledge to support a decision-making process).

#### What

Depending on *who* is receiving an explanation and *why* they require it, an explanation may aim to clarify either the internal processes of a system (i.e., how it specifically relates inputs to outputs) or its general behavior (i.e., input/output relationships only) and the explanation may need to be provided at the global (i.e., explains the entire model or system) or instance (i.e., explains a single prediction) level. The target and level of the explanation can generally be determined by the type of explanation the user is seeking. Lim et al. [16] provide a useful taxonomy of explanation types based on the intelligibility query they aim to answer: 1) “input” explanations, which provide information on the input values being used by a system; 2) “output” explanations, which provide information on specific outcomes/inferences/predictions; 3) “certainty” explanations, which provide information on why an expected output was not produced based on certain input values (i.e., contrastive explanations, counterfactuals); 4) “why” explanations, which provide information on how a system obtained an output value based on certain input values (i.e., model traces or complete causal chains); 5) “why not”/“how to” explanations, which provide information on why an expected output was not produced based on certain input values (i.e., contrastive explanations, counterfactuals); 6) “what if” explanations, which provide information on expected changes in output based on certain changes in the input (i.e., explanations that permit outcome simulations); and 7) “when” explanations, which provide information on which circumstances produce a certain output (i.e., prototype or case-based explanations). It should be noted that these taxonomy categories are not mutually exclusive (e.g., it is possible to provide an “input”/“output”/“certainty”/“why not” explanation). As discussed briefly in Wang et al. [15], user cognition and needs may also require explanations to be supported by additional information such as source data (e.g., raw data the model was built from), supplemental data (e.g., data not included in the modeling process but is relevant to the situation or context), and training materials (e.g., information on model development or explanation interpretation).

#### How

Summarizing and expanding upon the work of Doshi-Velez and Kim [18], the presentation of an explanation can generally be summarized using four main categories: 1) the unit of explanation, or the form or the cognitive chunk being processed (e.g., raw features, feature summaries, images, or instances); 2) the organization of the explanation units, or the compositionality and relationship between the units, which may include groupings, hierarchical or relational organizations, or summary abstractions (e.g., a free text summary of a combination of units); 3) the dimensionality, or processing size/levels of explanation information, which may include the overall size of an explanation or interactive exploration options; and 4) the manner in which information is represented, which includes the vocabulary, data structures, and visualizations used to express information. The specific choices in each of these four main categories will be determined by the target user for an explanation (i.e., *who*) and the environment in which it is being provided (e.g., *when/where*).

#### Scope of framework

The proposed framework aims to outline a general approach to designing user-centered displays of explanation that can be applied to existing explanation approaches and to the development of new approaches. Therefore, specific design suggestions (e.g., a specific explanation approach) are not included and examples are not meant to be comprehensive. Additionally, the framework was developed for empirically-based predictive models, or data-driven models based on statistical associations that aim to minimize prediction error. It is not intended to be used for explanatory models, or theory-driven models that aim to test causal relationships between variables and that may be used in prediction tasks. Similarly, our framework is intended to address model explainability and not causability, defined by Holzinger et al. [22] as follows:

*Explainability:* “in a technical sense highlights decision-relevant parts of the used representations of the algorithms and active parts in the algorithmic model, that either contribute to the model accuracy on the training set, or to a specific prediction for one particular observation. It does not refer to an explicit human model”.

*Causability:* “the extent to which an explanation of a statement to a human expert achieves a specified level of causal understanding with effectiveness, efficiency and satisfaction in a specified context of use”.

Therefore, when we refer to an “explanation display”, we are referring to a graphical display and related user interface designed to promote user understanding of factors contributing to a classification from a machine-learning model. Similarly, “explanation design” refers either to the process of creating such a display, or the elements included in such a display. We refer readers to Holzinger et al. [22] for more in depth discussions on explainability vs. causability.

## Methods

We provide a brief overview of the PICU in-hospital mortality risk model and utilize the proposed framework to suggest explanation displays for the model. Finally, we describe the methodology employed to conduct focus groups to solicit pediatric critical care provider feedback on the proposed displays.

### PICU in-hospital mortality risk model

Our motivating example was a mortality risk prediction model for PICU patients. As the main purpose of this work was to explore the potential benefit of user-centered explanation displays for the model, we utilized a small, readily available dataset and made no attempt to learn a best performing model. The Institutional Review Board (IRB) of the University of Pittsburgh approved the use of this data for the purposes of this work (PRO17030743).

The dataset included all discharged patients with a PICU admission at the Children’s Hospital of Pittsburgh (CHP) between January 1, 2015 and December 31, 2016. Variables included demographic information (age, sex, race), hospitalization data (time of admission and discharge), outcome data (discharge disposition and deceased date), assigned diagnoses, recorded locations, mechanical ventilation information, physical assessment measurements (vital signs, pupil reaction results, and Glasgow Coma Scale [23] measurements), and laboratory test results. The target outcome to predict was in-hospital mortality, defined as an encounter with a recorded deceased date that occurred on or prior to the recorded discharge date. The goal was to use all data collected prior to the time of the prediction to predict in-hospital mortality 24 h prior to the event. For death cases, this included all data collected up to 24 h prior to death; for control cases all data collected prior to discharge were included.

Data cleaning procedures included standardizing values for categorical data (e.g., race, diagnoses), grouping laboratory test and vital sign values measured by more than one technique (e.g., invasive/non-invasive blood pressures), removing text and invalid characters (e.g., “<”, “>”) from numeric results, and dropping invalid and duplicate test results. Features were defined for non-temporal (age, sex, race, length of stay, mechanical ventilation information, recorded locations, diagnoses) and temporal data (physical assessment measurements, laboratory test results). The final feature set included 422 features (see Additional file 1). Numerical features were discretized using the minimum description length criterion discretization method, [24] which accounts for class information (e.g., in-hospital mortality status) when defining discretization bins. After discretization, a “missing” category was introduced to each feature to account for missing values.

Using encounters from 2015 as training data and encounters from 2016 as testing data, we trained and evaluated several different models using a variety of feature selection techniques (information gain, correlation-based feature subset selection [25]) and learning algorithms (logistic regression, random forest, Naïve Bayes, support vector machine). We proposed explanation displays for the highest performing model, which was a 100-tree random forest model using features that had non-zero information gain scores, i.e., features that exhibited at least some predictive value. The model included 146 features and exhibited an area under the receiver operating characteristic curve (AUROC) of 0.94 and an area under the precision-recall curve (AUPRC) of 0.78.

### Proposed explanation displays

**Fig. 2:**
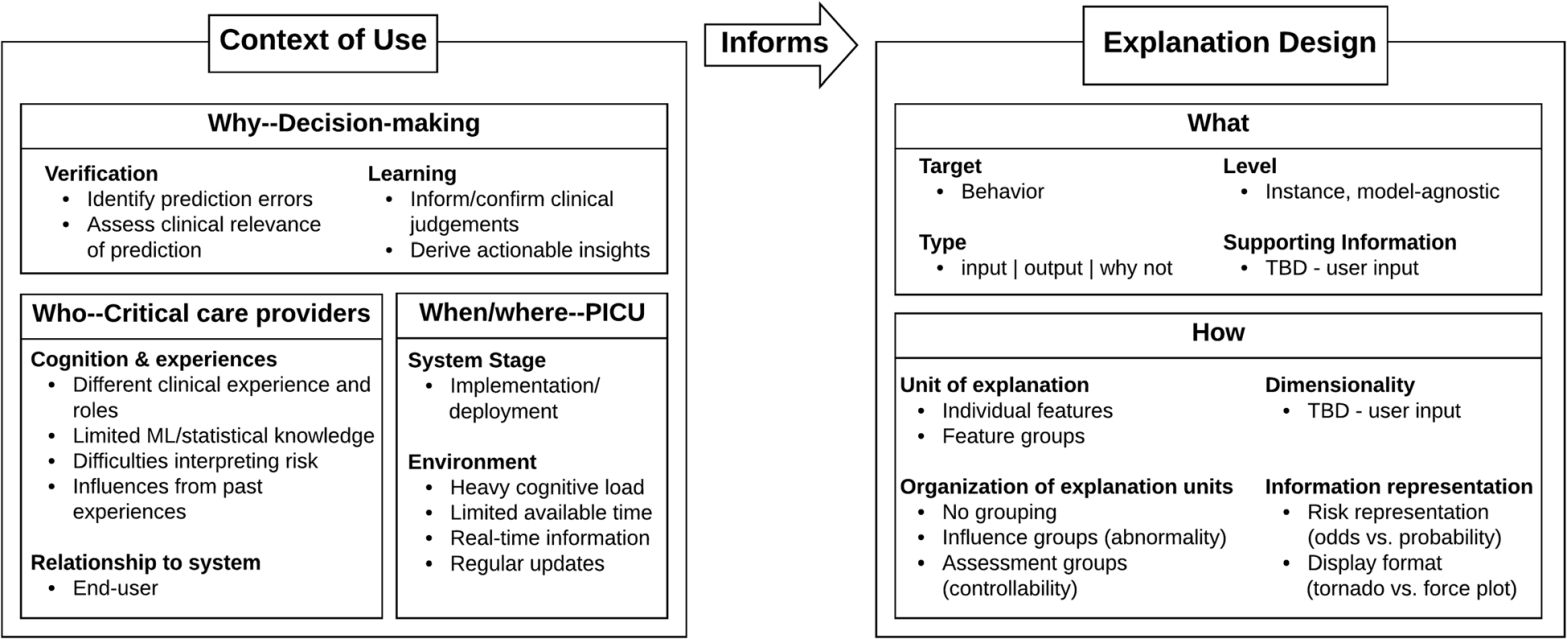
Summary of an initial context of use and a possible space of explanation designs. Note that any supporting information needs or dimensionality preferences would be determined based on input from users

We applied our proposed conceptual framework to define an initial context of use and identify promising design requirements for explanations of the PICU in-hospital mortality risk model. Figure 2 summarizes initial answers for each target question in the framework. These answers were derived from our prior experiences in developing predictive models as well as from an informal review of the literature on interpretable ML, social science work on human explanation and medical decision-making, HCI, information visualization, CDSS (specifically barriers, facilitators, and provider perceptions), and predictive models evaluated by providers or implemented in practice. As we anticipated that input from clinical users would be necessary to identify needed supporting information and dimensionality preferences, these fields were labeled as “TBD”.

We focused specifically on using the predictive model as a tool to support clinical decision-making in the critical care setting. As summarized in Fig. 2, explanations for the PICU in-hospital mortality risk model would likely be used by critical care providers who: 1) have varying clinical experience and limited knowledge on interpreting risk information from statistical and ML models (*who*), 2) work in a cognitively demanding, time-constrained environment where access to updated, real-time information is critical (*when/where*), and 3) would likely be seeking explanations to assist in verifying individual predictions from the model and learning information that can assist in decision-making (*why*). The answers to the *who* and *why* target questions suggest that a successful explanation design might contain (*what*) contrastive explanations (type: “why not”) for individual predictions (level: instance) that demonstrate which inputs push the prediction toward one output over another (target: behavior).

The aforementioned design requirements can be met by existing post-hoc explanation approaches that provide instance-level explanations based on feature influence values. Utilizing a model-agnostic explanation approach (i.e., an explanation approach that is not tied to any specific model or algorithm) would provide further benefit, as it allows the predictive model to adapt over time with minimal changes to the explanation display. However, the literature provides little to no insight on what supporting information (*what*) and presentation methods (*how*) would be effective for model-agnostic, instance-level, explanation approaches based on feature influence methods. Inquiries with critical care providers were required to validate the appropriateness of these explanations and understand what supporting information and presentation methods would contribute to a successful explanation display.

To facilitate these discussions, we proposed preliminary explanation displays for predictions from the PICU in-hospital mortality risk model. To generate model-agnostic, instance-level explanations of feature influence for the PICU in-hospital mortality risk model, we choose to utilize the Shapley additive explanations (SHAP) algorithm [26, 27]. We developed five low-fidelity prototype displays for the SHAP explanations, utilizing a variety of design options for the presentation of an explanation (*how*). Design options and rationales are described in Table 1.

**Table 1:** Design options and rationales for main factors to consider for explanation presentation

Factor	Design Options	Rationale
Unit of explanation	Individual features	Lower information granularity can reduce cognitive load and processing time. Evidence supports the use of lower information granularity for non-AI/ML experts via feature groupings or extractions [28]. We can group features by laboratory test/vital sign.
	Feature groups	
Explanation unit organization	None	Explanations including causes that are abnormal or controllable (i.e., modifiable) might be preferred [11]. Feature influence on risk might differ in abnormality (e.g., feature that increases risk might be considered abnormal). Assessment type groups might differ in controllability (i.e., laboratory tests are modifiable, demographics are not).
	Influence groups	
	Assessment groups	
Dimensionality (size & granularity)	Static	Dimensionality can be reduced through information removal (e.g., reducing explanation size) or aggregation (e.g., reducing explanation granularity). The desired dimensionality of an explanation may vary by individual and prediction, [29, 30] suggesting that interactive control over dimensionality could be beneficial. Examples include control over the granularity of explanation units and size (e.g. number of explanation units).
	Interactive	
Risk representation	Probability	Critical care providers should be comfortable with the risk representation format. Risk information in feature influence explanations has been previously reported in terms of odds and probability, [31, 32] but provider preferences on these representations are unknown.
	Odds	
Explanation display format	Force plot	Visual representations of risk information may facilitate comprehension of risk [33]. Tornado plots and custom visualizations called force plots have been used for feature influence explanations, [31, 32] but the effectiveness of these visualizations has not been validated in user studies.
	Tornado plot	

**Fig. 3:**
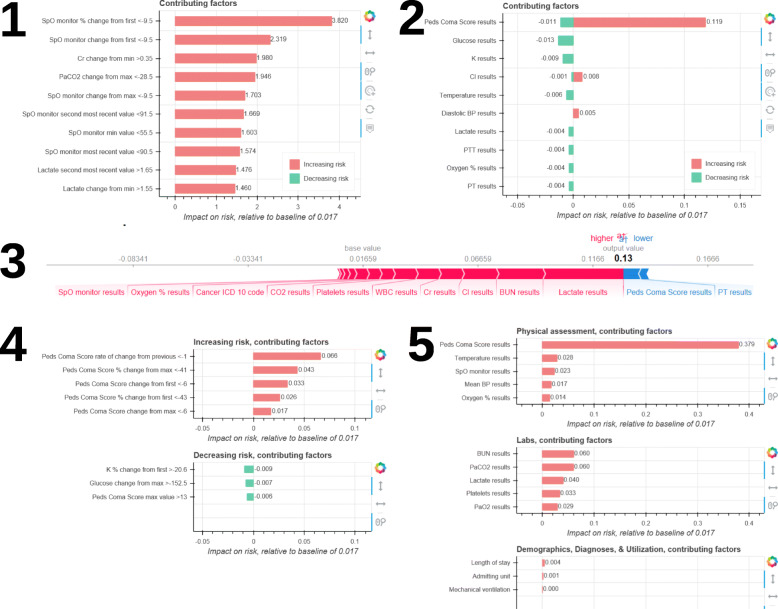
Prototypes of explanation displays utilizing different design options. Design options used in each prototype are listed as follows: a) unit of explanation, b) organization of explanation units, c) dimensionality, d) risk representation, and e) explanation display format. Prototype 1 options: a) individual features, b) none, c) interactive explanation size, static explanation unit granularity, d) odds, e) tornado plot. Prototype 2 options: a) feature groups, b) influence groups, c) interactive explanation size, interactive explanation unit granularity, d) probability, e) tornado plot. Prototype 3 options: a) feature groups, b) influence groups, c) static explanation size, static explanation unit granularity, d) probability, e) force plot. Prototype 4 options: a) individual features, b) influence groups, c) interactive explanation size, static explanation unit granularity, d) probability, e) tornado plot. Prototype 5 options: a) feature groups, b) influence groups and assessment groups, c) interactive explanation size, interactive explanation unit granularity, d) probability, e) tornado plot

**Fig. 4:**
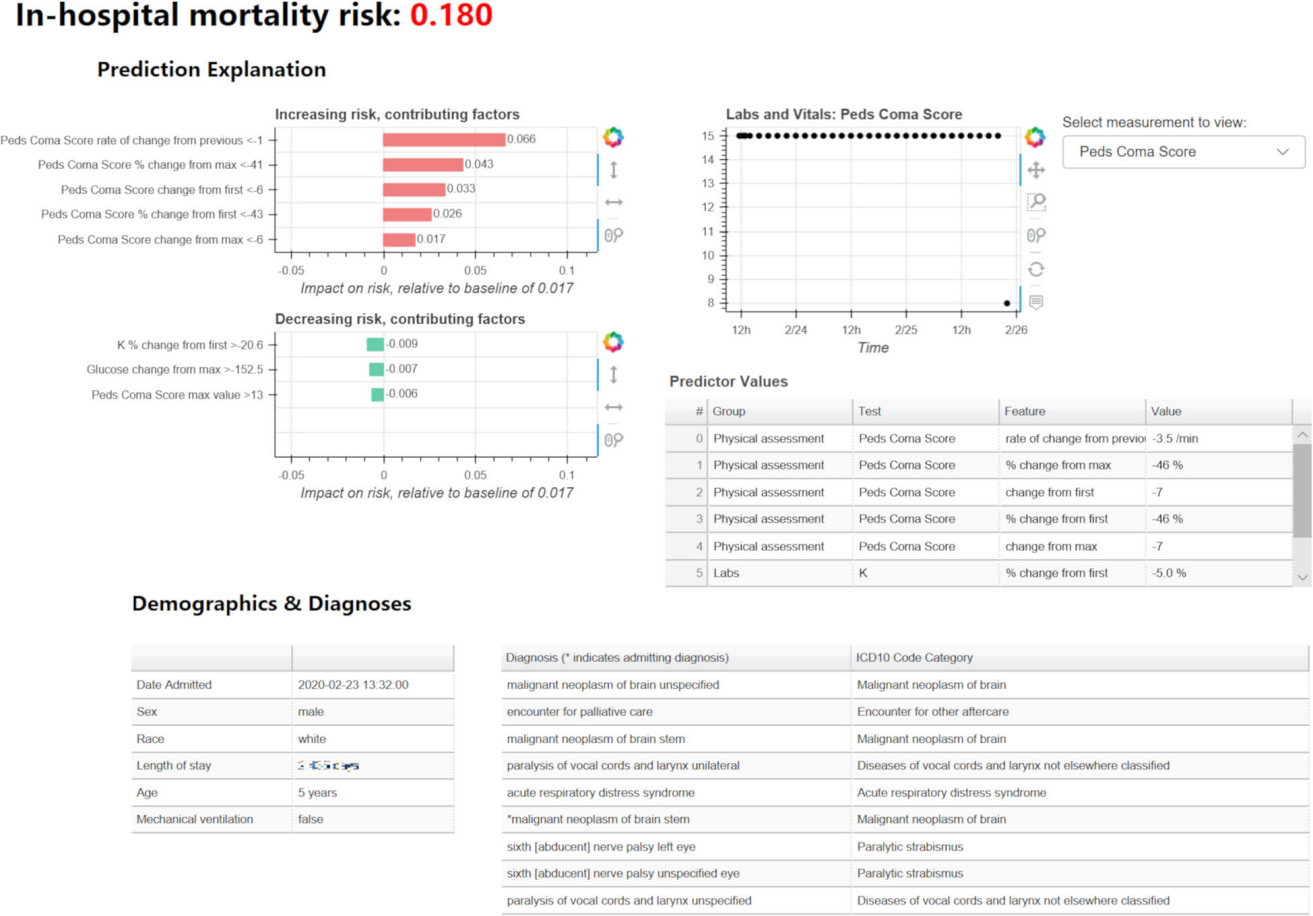
Example of complete prototype explanation display with supporting information. In addition to the explanation plot and model prediction (top left), each prototype included demographic information (bottom left), a list of current diagnoses (bottom right), a table of raw values of the features used in the model (middle right), and an interactive plot where the raw values of time series data from laboratory tests and vital signs could be viewed (top right). Please note that length of stay has been redacted to protect patient privacy

Each prototype included the predicted risk of mortality from the PICU in-hospital mortality risk model, an explanation for the predicted risk from the SHAP algorithm, and supporting information to assist in interpreting the risk and explanation. Figure 3 depicts the explanation portion and design options used in each prototype. Prototypes with feature groups and tornado plots also included an interactive hover-box option to view the individual level features within each group (i.e., modifiable granularity of explanation unit). Figure 4 describes and provides an example of the supporting information included in each prototype. SHAP explanations were generated using the Python shap package version 0.27.01 [34] and prototypes were generated as interactive HTML pages using the Python bokeh graphing package, version 1.0.4 [35].

### Focus groups to refine explanation display

We conducted focus groups with critical care providers to solicit feedback on the proposed explanation displays for the PICU in-hospital mortality risk model. Findings from the focus group were used to inform a final user-centered explanation display for predictions from the model. This study was reviewed and approved by the IRB at the University of Pittsburgh (STUDY19020074).

#### Setting and participants

Focus groups were conducted at CHP during March 2019–June 2019. A convenience sample of pediatric critical care providers of differing clinical expertise (e.g., nurses, residents, fellows, attending physicians) was recruited through professional connections of one of the authors (CMH).

#### Procedures and data collection

We conducted three focus group sessions, each ~ 1.5 h in length and comprising 5–8 participants. Each participant attended only a single focus group session, during which they were asked to complete three activities: 1) a guided group discussion about their perceptions of the PICU in-hospital mortality risk model based on information about its development; 2) a guided group review and critique of the five display prototypes; and 3) a questionnaire to indicate preferred design options. A focus group script and question guides were developed and followed for each session. See Additional file 2 for copies of the question guides for the group discussions. Focus group sessions were moderated by 1–2 researchers and a separate researcher took notes during each session. All sessions were audio-recorded.

#### Data analysis

Audio recordings of the sessions were transcribed verbatim and analyzed using descriptive coding [36]. One analyst (AJB) developed an initial codebook based on concepts and definitions from the proposed framework. Additional codes were included to subjectively capture participant perceptions of the credibility, utility, and usability of the model, which have been shown to influence adoption of predictive models in practice [37]. The analyst then applied the codes to the transcripts, refining definitions and adding codes to more finely represent the participants’ responses. A second analyst (HH) used the codebook to independently code one session transcript. The two analysts discussed coding differences to resolve disagreements and achieve consensus on a final codebook (see Additional file 3). The first analyst (AJB) then recoded all transcripts. Session notes recorded by the researchers were not coded, but were used to assist in coding and interpretation. QSR International’s NVivo 12 software [38] was used to assign and organize codes. This analysis was intended to identify findings related to each of the target questions in the proposed framework. Findings from the coding process were analyzed in conjunction with questionnaire responses to summarize findings about the context of use and explanation design preferences, identify perceived influences on critical care provider perceptions of the PICU in-hospital mortality risk model, and suggest a final user-centered explanation display for predictions from the model.

## Results

Twenty-one critical care providers participated in the focus group sessions. We describe the participants’ perceptions of the proposed explanation displays and present a final user-centered explanation display.

### Perceptions of participants on context of use

Table 2 summarizes focus group participants’ perceptions regarding context of use and summarizes our perceptions of participant comments regarding the credibility, utility, and usability of the PICU in-hospital mortality risk model. See additional file 4 for specific findings for each of the target questions related to context of use (*who, when/where, why*) with supporting quotes.

**Table 2:** Perceptions of focus group participants on context of use and perceived influences on model perceptions

User goal (why)	User characteristic (who)		Desired information	Positive (+) and negative (−) influences on perceptions
Verification	Predictive modeling knowledge	Detailed	Predictive performance Alignment with domain knowledge Comparison with existing models Modeling processes	Credibility + high predictive performance + predictions that aligned with clinical knowledge - influential outliers or data errors - counterintuitive risk factors - model limitations
		Basic	Predictive performance Alignment with domain knowledge	
Learning	Clinical role	Physician	Obtain patient insights: Prioritization Assessment of status Highlight patients/info of concern	Utility + training on use/interpretation - clinically irrelevant information
		Nurse	Actionable information Alerts to changes Information to intervene or justify consult	Usability + appropriate alerts - high cognitive effort or attention - large time investments

Providers sought explanations for two main reasons (*why*), with two main provider characteristics (*who*) influencing explanation information needs. Our participants wanted to use explanations to verify model information and assess model credibility. These explanation needs were influenced by the level of predictive modeling knowledge of a provider. While providers were concerned about the predictive performance of the model and how well its information aligned with domain knowledge, providers with more detailed knowledge of predictive modeling also wanted information about model development processes and how the model compared to similar existing models, such as the Pediatric Risk of Mortality-IV score [39]. High predictive performance and alignment with domain knowledge were viewed favorably, while limitations in the modeling process (e.g., not accounting for feature correlations) and predictions based on outliers, erroneous data, or counterintuitive risk factors were viewed unfavorably.

Healthcare providers also wanted to use explanations to extract knowledge or learn from the model, which related to perceptions of model utility and usability. These explanations needs were mainly influenced by the clinical position of the provider and factors relating to work environment (*when/where*). Specifically, participating physicians wanted explanations to obtain insights that would help them prioritize patients, assess patient statuses, and identify high-risk patients and information of concern. Nurses sought directly actionable information, such as alerts to important changes in patient status and information to assist in intervention or in justifying a request for a physician consult. Regardless of clinical role, information seen as clinically irrelevant (e.g., a high-risk prediction driven by a low Glasgow Coma Score measurement for a sedated and paralyzed patient) was viewed unfavorably. Participants raised concerns about the need for training to ensure proper interpretation and use of the model. Concerns about appropriate alerts (i.e., relevant and at proper time) and limiting the time and attention required to use the model suggested that perceptions of model usability might be influenced by fit with clinical workflow.

Finally, while providers did not generally seek explanations with the goal of improving the model, we found that providers used the information provided in the prototypes to suggest improvements. Suggestions for improvement mainly involved incorporating domain knowledge into the model, such as including additional relevant predictors (e.g., medications) and defining normal ranges for variables (e.g., setting age- or patient-specific baselines). Participants also suggested improving model utility by examining alternative prediction targets, such as morbidity, disease-specific mortality, and event-specific mortality (e.g., mortality as a result of a cardiac event).

### Explanation design preferences

**Fig. 5:**
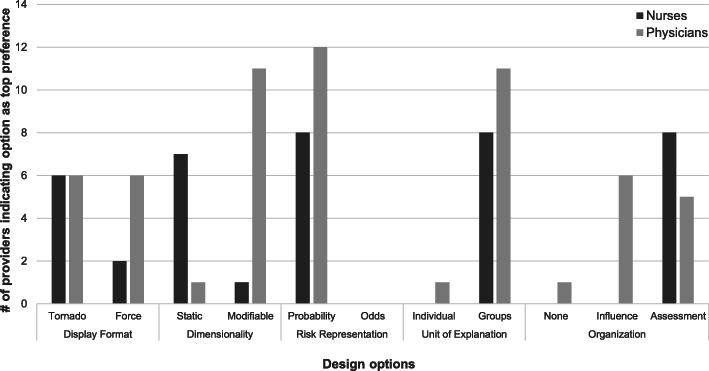
Provider preferences on prototype design options by clinical role

Table 3 summarizes provider preferences for explanation display content and design. See additional file 5 for specific findings for each of the target questions related to explanation design (*what, how*) with supporting quotes. Figure 5 summarizes healthcare provider preferences.

**Table 3:** Explanation design preferences

Desired content (what)	Benefits	Preferred design (how)
Explanations	Help assess model credibility and utility	Risk expressed as percent probability High-level information with details available on demand Interactive options to support different displays/organizations for various users
Table of raw feature values	Interpret discretized features Examine trend-based features	Directionality for trend-based features Simpler terminology
Time-series data plot	Investigate suspicious values Assess trends and baselines	Multiple plots Highlight points related to features Auto-population of data
Contextual information	Clinically meaningful interpretation Context for risk prediction	Providing clinical context information Prominent display of baseline risk Inclusion of risk trends

All providers preferred explanation displays that reduced information processing effort, such as expressing risk as percent probability and aggregating information (e.g., grouping all features related to a measurement such as blood pressure instead of showing individual features). Differences in clinical roles and individuals led to mixed preferences on other design options. Nurses wanted minimal, actionable information and tended to prefer simpler, static explanations organized by assessment groups. Physicians preferred more dynamic explanations. To support efficient data exploration for different users, providers suggested interactive explanations in which additional details could be obtained on demand (e.g., viewing individual risk factors in a group) and users could have control over how they organize and view explanation content.

Supporting information in the prototypes was vital to provider interpretation of the predictions and explanations. Providers found the table of raw feature values helpful when examining trend-based features and interpreting the discretized features used in the explanation. For example, if the most predictive feature in the explanation was “Cr change from min > 0.35” (i.e., the most recent value of creatinine has increased more than 0.35 since the minimum value), the exact amount of the increase, the current value, and the minimum value were all described by providers as helpful information. Providers' suggestions for improving the usability of the prototypes included the addition of indicators of the directionality of trend-based features in this table (e.g., trend has increased) and the adoption of simpler terminology for trend-based features in the explanation plot (e.g., “Cr has increased since minimum value”). Providers frequently used the time-series plot of raw laboratory tests and vital sign data to investigate suspicious values (e.g., outliers or errors), assess trend-based features, and determine patient baselines. Providers suggested improving the usability of these plots by including multiple plots to view and compare data, highlighting the specific points used to derive features in the model, and auto-populating data on these plots when an element was selected in the explanation plot or table of raw feature values.

Although the prototypes contained only minimal contextual patient information, those details were considered to be important in assessing the clinical relevance of a prediction. Interventions were seen as particularly important. For example, a high-risk prediction driven by a low Glasgow Coma Score measurement would be of less concern to a provider if they knew the patient was sedated and paralyzed at the time of the measurement. Informants also noted that baseline risk levels provided important context when interpreting predictions (e.g., a predicted risk of 40% was more concerning if they knew the baseline risk was 2%), and suggested moving this information to a more prominent display location. Additionally, providers suggested that including risk trends would improve model utility, as a change in risk might be more clinically relevant than a single predicted risk (e.g., a patient that has had a high predicted risk for several days might be of less concern than a patient with a lower predicted risk that recently increased).

Participants’ interest in contextual information extended to the exploration of clinician responses to elevated risk. Several participant comments in our focus groups indicated a desire for actionable information, including ways to mitigate risk.

### Proposed user-centered explanation display

**Fig. 6:**
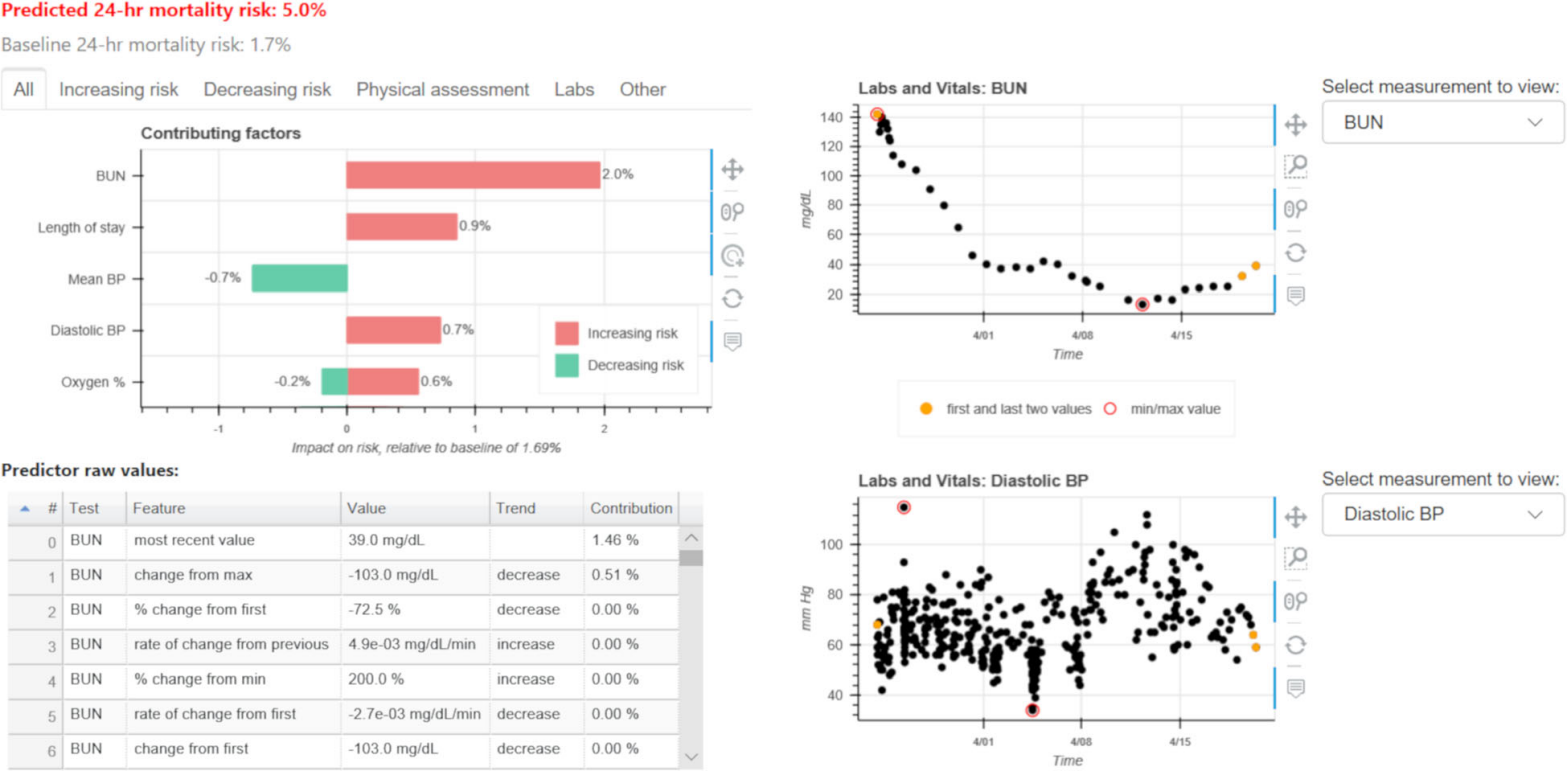
Final user-centered explanation display. The predicted risk and baseline risk are displayed in percent probability at the top of the figure. The explanation plot (top left) uses feature groups as the unit of explanation, but has hover-box capability to view individual features within each feature group. In the hover-box, trend-based features are summarized by trend direction (e.g., “Cr has increased since minimum value”). The plot includes interactive controls to view additional feature groups (e.g., scrolling down the explanation plot) and view different sets of feature groups (e.g., view laboratory test feature groups). The table of raw feature values (bottom left) includes the description, value, and contribution to the predicted risk for each individual feature. This table also includes the trend direction for trend-based features. The time-series plots to display raw values of laboratory test and vital sign data (right) highlight the points used to compute features and include interactive controls to zoom in on and select regions of data. These plots also have hover functionality that can be used to show the value and time of a specific point. To facilitate data exploration, interactivity is linked across plots and tables (e.g., selecting a predictor on the explanation plot will highlight it in the raw feature table and load the appropriate laboratory test/vital sign in the time-series plot)

Based on the findings from the previous sections, we proposed a final user-centered explanation display for individual predictions from the PICU in-hospital mortality risk model. The final user-centered display is shown and described in Fig. 6. It should be noted that although providers exhibited mixed preferences on the explanation display format, we selected the tornado plot as some providers had found that the force plot was confusing to interpret. The final display also excluded the contextual information requested by participants (e.g., interventions), as we assume this display would be integrated into an electronic health record (EHR) system where such information would be readily available.

## Discussion

The premise underlying this work is that explanation displays designed to meet the information needs of healthcare providers might be a means of making ML-based clinical predictive tools that are more acceptable to clinicians.

Toward that end, we proposed a qualitative research framework of explanation design that addresses all context of use factors relevant to utilizing a predictive model in clinical practice. This framework provides a starting point for exploring the impact of design choices, specifically considering the impact that the environment may have on the information needed in an explanation display. We demonstrated an application of the framework by utilizing insights from the literature and prior experiences to suggest explanation displays that were then refined through healthcare provider feedback.

In addition to the proposed framework, our work contributes some perspective on the design of displays capable of effectively communicating predictive model risk information to healthcare providers. Focus group comments suggested that providers found the prototype displays of the SHAP explanations useful in assessing the credibility and utility of a prediction from the model (i.e., comparing the influential risk factors to domain knowledge to determine if the prediction seemed reasonable and clinically relevant). This suggests that model-agnostic, instance-level explanation approaches based on feature influence methods may be a viable approach to explaining model predictions in a way that is both comprehensible and useful to healthcare providers. Although other studies have utilized these approaches to explain predictive models in healthcare, [4, 31, 40] to the best of our knowledge this is the first study to verify that these explanations would be positively received by healthcare providers.

Participants in our focus groups were enthusiastic about the ability to visually assess which risk factors were contributing most to an individual’s predicted risk. This finding is consistent with claims in the literature that visualizations of risk information for individuals can improve healthcare provider interpretation and acceptance of predictive models [33, 41]. Additionally, our informants suggested that providing the appropriate contextual information was vital to provider interpretation of risk. In particular, access to raw patient data (e.g., laboratory values, vital signs, interventions) was seen as useful for assessing the clinical credibility and utility of predictions and explanations. This finding is consistent with results from studies by Wang et al. [15] and Jeffery et al., [42] both of whom also found that providers utilized raw patient data when working with probability-based decision support systems to verify predictions and to integrate them with their clinical knowledge.

In addition to raw patient data, baseline risks and risk trends were identified by several nurses in our focus groups as important information necessary to assess the clinical relevance of a risk prediction. More specifically, a change in risk from a patient-specific or population baseline was deemed more clinically relevant than a single risk prediction. This finding is consistent with results from Jeffrey et al., [42] who also found that nurses wanted to see risk trends when using probability-based CDSS. This suggests that displaying risk trends for individual patients might improve the perceived utility of a risk prediction model. As we intended to design explanations for single-risk predictions from the PICU mortality risk model, we did not incorporate risk trends into our final user-centered explanation display. However, future work should consider examining how the display of risk trends in conjunction with individual prediction explanations affects provider perceptions of model utility.

The focus group sessions also suggested that interactive risk explanation tools would be beneficial. By supporting “drill-down” to additional details, these details would support a range of information needs, as clinicians interested in seeing details could “zoom in” from overviews designed to minimize information overload. Interactive explanations would also facilitate inclusion of multiple explanation types into a single explanation display, such as incorporating “what if” type explanations that allow users to simulate how changes in model inputs affect predicted risk. The need for integrating multiple explanation types into a single explanation display was previously mentioned by Wang et al., [15] who found that providers utilized a variety of different explanation types to support various reasoning processes when diagnosing patients. Our proposed framework could support the exploration of combinations of explanations to support healthcare provider explanation needs in various tasks.

Our previous experience suggested that clinical predictive tools were potentially less likely to be used if clinicians did not have a means of responding to predictions of high risk of adverse outcomes. Comments from our focus group were consistent with this experience, as several participants indicated that they would like the explanation displays to emphasize actionable information. This suggests that providing recommended actions might be beneficial. This approach has been suggested in prior literature as a way to improve model acceptance [20, 43]. Developing clinical recommendations for data-driven models can be challenging, as reasoning processes of statistically-driven models often do not correlate with clinical knowledge. However, some providers in our focus groups were able to translate the explanation displays into clinically relevant explanations (e.g., a set of risk factors indicating multi-system organ failure in a high-risk patient) that could potentially be mapped to recommended actions. Accommodating provider requests for more specific prediction outcomes (e.g., disease-specific or event-specific mortality) might have further improved their ability to use the explanation displays to identify potential avenues for risk mitigation and develop recommended actions. Explanation displays could be beneficial tools in developing recommended actions to pair with data-driven model predictions.

One could argue that there may be scenarios in which explanations are not required at all. For example, if a model was able to achieve perfect performance on a prediction task or when it is obvious whether the model correctly predicts the outcome (e.g., image classifications that can be verified by visual inspection), explanations might be considered unnecessary. Often, the argument for explanations centers around instilling user trust in the model; however, Elish [10] argues that trust is not predicated on model interpretability, and can instead be developed by involving stakeholders throughout the model development process. Even if explanations are not required to verify model accuracy or instill trust in a model, they may still prove valuable by providing actionable information. For a general mortality model like the one in this study, a provider’s ability to intervene would depend on knowing why the model predicts that a specific patient will die. Thus, an explanation might still be valuable in this context. The need for explanations for predictive models in healthcare will be context-dependent and should be discussed with stakeholders in the early stages of model development.

This study has some limitations that should be addressed in future work. Our focus group sessions only included providers from a single institution. Our claims regarding explanation design in healthcare should be validated in other groups of providers across a multitude of institutions. To maximize the number of participants in our study, we scheduled sessions based on participant availability which resulted in participant groups that lacked diversity in clinical role (e.g., all nurses attended one session). We also only utilized focus groups to solicit healthcare provider feedback on our proposed explanation displays. We may have missed important insights that would arise from discussions between providers in different roles and under different approaches to user-centered design, such as a participatory design exercise like the one utilized by Jeffrey et al. [42] Future applications of the proposed framework should explore alternative approaches to soliciting user feedback on explanation displays, including the use of psychometrically-validated studies for assessing attitudes toward the potential utility of the explanations and preferences regarding the explanation interfaces. The recently proposed System Causability Scale (SCS) [44], designed to measure explanation quality and based on the notion of causability, might be used to evaluate and compare the utility of explanations. Another limitation of this study is that we do not report on a validation of the final user-centered explanation display for the PICU mortality risk model. Results of a completed study of the impact of the user-centered explanation display on impact provider acceptance and use of the PICU mortality risk model in decision-making processes will be presented in a forthcoming publication.

Providers’ reactions to the SHAP explanations suggest another direction for future work. Providers used the explanation displays to identify possible improvements to the clinical credibility and utility of the PICU in-hospital mortality risk model, suggesting that instance-level explanations could be used to foster improved communications between model developers and clinical domain experts. Potential designs might include interfaces that providers use to indicate when and why individual predictions might be inaccurate. This feedback could then be used to inform refinements to the predictive models and explanations. This approach is supported by Holzinger et al. [22], who state that AI systems not based on causal models (e.g., statistical models) are limited by their inability to understand context and reason about interventions. They propose interactive ML interfaces as tool to incorporate human expertise into probabilistic models as an intermediate step leading to causal models [44]. Incorporating healthcare provider feedback and knowledge into models has also been shown to improve acceptance of predictive models in practice, [9, 10] providing further support for the need to develop interactive interfaces. Future studies will apply our framework to inform the design of interfaces that facilitate these interactions.

## Conclusions

We proposed a framework for designing user-centered explanation displays for ML models and demonstrated its use in the design of an user-centered explanation display for predictions from a PICU in-hospital mortality risk model. Focus group discussions with healthcare providers confirmed that the proposed model-agnostic, instance-level explanations of feature influence were viable approaches to explaining model predictions to healthcare providers and informed the iterative redesign of an explanation display. This work also revealed several insights about the effective communication of predictive model risk information to healthcare providers. It is our hope that findings from this work will facilitate conversations with healthcare providers about the development, deployment, and continuous improvement of ML-based tools that can promote positive changes in clinical practice.

## Supplementary information

**Additional file 1.** Description of features generated to learn PICU mortality models. Table containing the name and definition of all temporal and non-temporal features derived from raw data and used to learn PICU mortality models.

**Additional file 2.** Focus Group Question Guide. List of questions and potential follow-up questions used to conduct focus group sessions.

**Additional file 3.** Descriptive Coding Final Codebook. Final codebook derived from descriptive coding process that contains codes used to analyze transcripts and derive insights from focus group sessions.

**Additional file 4.** Perceptions of participants on context of use with supporting quotes. Table of findings with supporting quotes for each target question under the context of use portion of the proposed framework. All findings were derived from the analysis of the focus group session transcripts.

**Additional file 5.** Participant preferences on explanation design with supporting quotes. Table of findings with supporting quotes for each target question under the explanation design portion of the proposed framework. All findings were derived from the analysis of the focus group session transcripts.

## Data Availability

The datasets analyzed in this study are available from the corresponding author on reasonable request.

## Abbreviations

AI: Artificial intelligence
AUPRC: Area under the precision-recall curve
AUROC: Area under the receiver operating characteristic curve
CDSS: Clinical decision support systems
CHP: Children’s Hospital of Pittsburgh
HCI: Human computer interaction
IRB: Institutional review board
ML: Machine learning
PICU: Pediatric intensive care unit
SHAP: Shapley additive explanations
