# Supplementary material for: A qualitative research framework for the design of user-centered displays of explanations for machine learning model predictions in healthcare
Source: BMC Med Inform Decis Mak. 2020 Oct 8;20:257. doi: 10.1186/s12911-020-01276-x (PMC7545557; doi:10.1186/s12911-020-01276-x)
Supplement: Supplementary file 1 — Additional file 1. Description of features generated to learn PICU mortality models. Table containing the name and definition of all temporal and non-temporal features derived from raw data and used to learn PICU mortality models. [file 12911_2020_1276_MOESM1_ESM.docx]

**Description of features generated to learn PICU mortality models**

| **Non-temporal features** | | |
| --- | --- | --- |
| *Feature Name* | *Definition* | |
| Age | Patient age in days | |
| Sex | Patient sex | |
| Race | Patient race | |
| Length of stay | Elapsed time between arrival date and time of prediction | |
| PICU admitting unit | Unit location immediately prior to first recorded visit to PICU | |
| Admitting diagnosis category | ICD10 category of admitting diagnosis code | |
| CPR flag | Presence/absence of pre-defined cardiac arrest diagnosis code | |
| Cancer flag | Presence/absence of pre-defined cancer diagnosis code | |
| Mechanical ventilation flag | Presence/absence of recorded ventilator event | |
|  | | |
| **Temporal features** | | |
| *Feature Name* | *Definition* | *Covered Tests/Measurements* |
| First value | Result with earliest timestamp in defined time-window (missing if <3 results) | Pupil reaction and all tests/measurements listed below |
| Second most recent value | Result with second most recent timestamp in defined time-window (missing if <2 results) |  |
| Most recent value | Result with most recent timestamp in defined time-window |  |
| Min value | Minimum result recorded in defined time-window | -Blood urea nitrogen (BUN)  -Chloride (Cl)  -Bicarbonate level (CO2)  -Creatinine (Cr)  -Diastolic blood pressure (diastolic BP)  -Glucose -Heart rate  -Potassium (K)  -Lactate  -Mean blood pressure (mean BP)  -Oxygen %  -Arterial partial pressure of -carbon dioxide (PaCO2)  -Arterial pressure of oxygen (PaO2)  -Cutaneous carbon dioxide tension (PcCO2)  -Glasgow coma scale (Peds coma score)  -pH  -Platelets  -Prothrombin time (PT)  -Partial thromboplastin time (PTT)  -Venous partial pressure of carbon dioxide (PvCO2)  -Systolic blood pressure (systolic BP)  -SpO2 bedside monitor (SpO monitor)  -Temperature  -White blood cell count (WBC) |
| Max value | Maximum result recorded in defined time-window |  |
| Change from previous | Most recent value – second most recent value |  |
| Change from min | Most recent value –min value |  |
| Change from max | Most recent value – max value |  |
| Change from first | Most recent value – first value |  |
| % change from previous | (Most recent value – second most recent value)/(second most recent value)*100 |  |
| % change from min | (Most recent value – min value)/(min value)*100 |  |
| % change from max | (Most recent value – max value)/(max value)*100 |  |
| % change from first | (Most recent value – first value)/(first value)*100 |  |
| Rate of change from previous | Slope between most recent value and second most recent value |  |
| Rate of change from min | Slope between most recent value and min value |  |
| Rate of change from max | Slope between most recent value and max value |  |
| Rate of change from first | Slope between most recent value and first value |  |
| # results w/ both pupils nonreactive | Count of pupil reaction results where both pupils were nonreactive | Pupil reaction |
| # results w/ one pupil nonreactive | Count of pupil reaction results where one pupil was nonreactive |  |
