# Supplementary material for: A qualitative research framework for the design of user-centered displays of explanations for machine learning model predictions in healthcare
Source: BMC Med Inform Decis Mak. 2020 Oct 8;20:257. doi: 10.1186/s12911-020-01276-x (PMC7545557; doi:10.1186/s12911-020-01276-x)
Supplement: Supplementary file 2 — Additional file 2. Focus Group Question Guide. List of questions and potential follow-up questions used to conduct focus group sessions. [file 12911_2020_1276_MOESM2_ESM.docx]

**Focus Group Question Guide**

| **Model Discussion** |
| --- |
| **Question Guide**   - *How would you feel about deploying these kind of predictive models into clinical practice?* - *What practical applications do you think these kinds of models could have in clinical practice?* - *Would you feel confident in the predictions provided by these kinds of models?*    - *What additional information about the model would you require in order to have confidence in its predictions?* - *Do you think you would use predictions from models like this? Why?* - *Apart from predicting other outcomes, how could these kinds of models be made more useful?* - *You may have noticed that not much information about the model or the underlying algorithm was provided. How might this information influence your perceptions of a model? What assumptions, if any, did you make about the model or underlying algorithm?* |
| **Mock-up Review** |
| **Individual Mock-up Question Guide**   - *How would you summarize why the model made this prediction?* - *Why might you be inclined to believe or disbelieve a prediction presented in this fashion?*   - *Are any predictors surprising or non-sensical?*   - *What information led you to belief/disbelief of the prediction?* - *What information is missing that might help you interpret this prediction more effectively or efficiently?*   - *Model performance? Confidence intervals for contribution values?*   - *Different grouping or order of predictors? Different number?* - *What information provided might you find useful in performing your job?*   **Mock-ups 1, 2, and 3 Comparison Question Guide**   - *What do you think of displaying risks as probabilities versus odds? Which do you prefer? Why?* - *What do you think of displaying individual predictors versus groups of predictors? Which do you prefer? Why?* - *What do you think of the tornado plot versus the force plot? Which do you prefer? Why?* - *What would you change about any of these displays?*   - *What changes would make a display easier to understand?*   - *What information or design elements do you think are missing?*   - *What information or design elements are not useful?*   **Mock-ups 4 and 5 Comparison Question Guide**   - *How does grouping predictors into plots change your opinion of displaying individual predictors versus groups of predictors?* - *What do you think of grouping predictors into multiple explanation plots?*    - *What alternative ways to group predictors can you think of?*   - *What is your preferred grouping, or would you prefer no grouping?* - *What would you change about any of these displays?*   - *What changes would make a display easier to understand?*   - *What information or design elements do you think are missing?*   - *What information or design elements are not useful?* |
