# Supplementary material for: A qualitative research framework for the design of user-centered displays of explanations for machine learning model predictions in healthcare
Source: BMC Med Inform Decis Mak. 2020 Oct 8;20:257. doi: 10.1186/s12911-020-01276-x (PMC7545557; doi:10.1186/s12911-020-01276-x)
Supplement: Supplementary file 3 — Additional file 3. Descriptive Coding Final Codebook. Final codebook derived from descriptive coding process that contains codes used to analyze transcripts and derive insights from focus group sessions. [file 12911_2020_1276_MOESM3_ESM.docx]

**Descriptive Coding Final Codebook**

| Name | Description |
| --- | --- |
| 1. Context of use--when & where | The environment in which the explanation will be used, which is often related to the stage of system development. Environment will dictate the available user time and cognitive capacity, the available technical resources, and the user’s perception of the system, which all may influence explanation design. This main category code is meant for organizational purposes only and should not be applied. |
| 1.1 Environment | Aspects of the environment that will affect how an explanation needs to be designed in order to support use within that environment. This parent code should only be applied when a participant comment falls within this parent category, but none of its children codes can be applied appropriately. |
| 1.1.1 Cognitive and time resources | Participant cognitive capacity and/or time availability to use the system in a specific environment. This includes comments about cognitive effort to process information in a given time frame (e.g., ease and speed of information processing, time restrictions, willingness to spend mental effort or time) and workflow or other environmental influences that imply a possible impact on cognitive capacity or time availability (e.g., task order, when/how/where to capture attention)  Example:  -Speed or ease with which knowledge can be obtained from system (e.g., faster synthesis of relevant information, familiarity with the way information is presented) |
| 1.1.2 Social and organizational influences | Any aspect of the social or organizational environment in which the system is being used that may impact system development, design, or application. This can include things related to participant workflow, organizational infrastructure (e.g., staffing procedures/challenges, patient triage/bed assignment procedures/challenges, education/training programs, financial policies), and social pressure/expectations.  Examples:  -Workflow, such as rounding practices, patient/colleague interactions, EHR interactions, etc.  -Staffing/triaging procedures, such as bed availability, staff availability, etc. |
| 1.1.3 Technical resources | Technical resources available (e.g., compatibility with existing systems, processing/memory constraints) when using the system in a specific environment. This can include limitations of pre-existing systems, difficulties with real-time data processing, and challenges in implementation and/or maintenance of the system. |
| 1.2 System stage | Design/information needs in a specific system stage (e.g., development, implementation, deployment). This code should only be applied when a different system stage may require a change in information/design needs.  Example:  -a participant mentions specific information which would assist in validating the predictive model (this may be an indirect reference to information/design needs in the development stage, which may differ from needs in the deployment stage) |
| 2. Context of use--who | User's cognition (e.g., knowledge, experience, capabilities, etc.) and the user’s relationship to the system at the time the explanation is being provided. A user may have several different relationships with the system over time, and thus their explanation needs may change with varying roles. This main category code is meant for organizational purposes only and should not be applied. |
| 2.1 Cognition & experiences | The knowledge, experience, capabilities, etc. of the user of a system. Three main categories of user cognition to consider include AI experts, domain experts, and lay persons. Of particular interest is any aspect of the user’s background knowledge or prior experiences that may bias their opinion of or attitude toward a new system. This parent code should only be applied when a participant comment falls within this parent category but none of its children codes can be applied appropriately. |
| 2.1.1 Background knowledge | Participant’s prior level of knowledge of ML/AI/predictive modelling concepts. Includes remarks/questions that suggest knowledge (or lack thereof) of predictive models (e.g., objective reference to known model, mentions of ML algorithms, model limitations/validity) or the model development process (e.g., cohort definition, data cleaning, feature engineering, training, evaluation, bias/overfitting, best practices). Not applicable to remarks/questions on presentation content (e.g., AUC, inputs) |
| 2.1.2 Prior experiences | Participant’s prior experience with using an ML/AI tool or another information system (e.g., EHR). This code is restricted for use when the participant expresses either a positive or negative opinion or attitude about the design, credibility, usability, or utility of the tool/system, and should not be used to code objective comparisons of tools/systems (e.g., comparing performance or data inputs, objective discussions on design and implementation). |
| 2.2 Relationship to system | The user's relationship to the system at the time the explanation is being provided. Main roles to consider can be engineer, developer, owner, end-user, data subject, and stakeholder. It should be noted that a user may occupy more than one role simultaneously. This parent code should only be applied when a participant comment falls within this parent category but none of its children codes can be applied appropriately. |
| 2.2.1 User perspective | How system design or system application might differ based on the user's current relationship with the system. This can include comments about design differences based on intended system application (e.g., developer vs end-user needs, different end-user information needs). This code should not be applied to design/application differences that would arise from variation in user cognition & experiences (e.g., background knowledge, thought processes) |
| 3. Context of use--why | User needs and goals that drive the need for an explanation. Four general reasons why explanations of intelligent systems are required include verification, improvement, learning, and compliance. Needs/goals will vary according to the who/when/where elements of the context of use. This main category code is meant for organizational purposes only and should not be applied. |
| 3.1 Compliance | Closely related to verification (3.4), this refers to any activities aimed at ensuring the system adheres to an established legal, moral, or other societal standard. This includes all comments on the system from an ethical, moral or legal/organizational policy standpoint. |
| 3.2 Improvement | Closely tied with verification (3.4), this covers activities related to improving system performance and efficiency. May include incorporating domain knowledge to reduce biases in or improve generalization of model, comparing/selecting models, and improving system response times. Includes suggested changes in data collection, data inclusion/exclusion, data processing, and target outcomes/definitions. Not applicable to comments about or suggestions to improve model utility (e.g. possible applications of current model information, post-hoc analyses such as distribution of risk scores across units, or tracking risk scores and outcomes of specific patients).  Examples:  -suggestions to explore predictions of an outcome other than 24-hr pediatric ICU mortality (e.g., time to event predictions, morbidity, ICU transfer, mortality in a specific patient population, etc.)  -suggestions to include additional data such as tests, staffing, comorbidities, bed location, etc.  -suggestions to improve data processing such as removing outliers, dropping bad values, defining normal ranges, adjusting for age/condition, etc. |
| 3.3 Learning | Remarks/questions indicating participant is seeking to gain knowledge or information from the system, including identifying new data patterns, generating/testing new hypotheses, and/or providing support for decision-making (e.g., provide supporting evidence for a decision, improve decision-making speed or accuracy, identifying actionable information such as courses of action or modifiable risk factors). |
| 3.4 Verification | A possible reason for requiring an explanation of an intelligent system. Includes examining how decisions/suggestions are made by the system to ensure it is operating as expected, which may include activities such as detecting biases, finding/debugging errors, and ensuring that system reasoning aligns with domain knowledge. This parent code should only be applied when a participant comment falls within this parent category but none of its children codes can be applied appropriately. |
| 3.4.1 Comparing against known model | Comparison of model to existing model/tool to validate some aspect of the system (e.g., credibility). Only applicable to remarks that compare objective metrics (e.g., performance, data collection & processing). Not applicable to participant opinions of existing models or preferences for system content & design (use "prior experiences" and "explanation design" category codes instead). Generally not applicable to comments related to system utility (use "Learning" category codes instead). |
| 3.4.2 Comparing model information to domain knowledge | Comparison of system information against clinical knowledge to verify some aspect of the model (e.g., credibility). This can include comments or questions about possible data biases, information validity, etc. Not applicable to remarks where participants are suggesting improvements based on clinical knowledge. Generally not applicable to comments related to system utility (use "Learning" category codes instead). |
| 3.4.3 Seeking information on model development processes | Remarks/questions seeking to validate any aspect of the model development and maintenance process (e.g., cohort definition, data sources, data collection/inclusion/exclusion, cleaning processes, feature engineering/selection, model learning process, evaluation, maintenance over time). Applicable only when participants make assumptions about or attempt to clarify/understand/question the model development/maintenance process and is not applicable to suggestions for improvement. |
| 4. Explanation design--how | Can generally be determined by the who and why questions of context of use, and refers to the way in which the content of an explanation is presented to a user. The presentation of an explanation can generally be summarized using 3 main categories: dimensionality, explanation unit granularity and organization, and information representation. This main category code is meant for organizational purposes only and should not be applied. |
| 4.1 Dimensionality | A main category to consider when designing an explanation. Refers to the processing size/levels of explanation information, which may include the overall size of an explanation or interactive exploration options. Should only be applied when a participant comment falls within this parent category but none of its children codes can be applied appropriately. |
| 4.1.2 Size & interactivity preferences | Preferences for the size and/or interactivity options in the explanation design. Applicable to preferences on interactivity/size options in mock-ups (e.g., plot hover and drop-down select capabilities, link between explanation plot and predictor table, scrollable list of predictors) and any suggestions for interactivity/size options not shown in mock-ups (e.g., interactions between visualizations, amount of information content, interactive explanation exploration options). |
| 4.2 Explanation unit & organization | Preferences regarding the granularity and organization of the explanation units. This includes preferences on the unit of explanation or predictor granularity (e.g., raw predictors, grouped/summarized predictors, increasing/decreasing or net contributions) and organization of the explanation units (e.g., order of display, location of increase/decrease contributions, grouping into different plots). Applicable to both remarks on mock-up options and suggested alternatives. |
| 4.3 Information representation | A main category to consider when designing an explanation. This includes the vocabulary, data structures, and visualizations used to express information. This parent code should only be applied when a participant comment falls within this parent category but none of its children codes can be applied appropriately. |
| 4.3.1 Data visualization preferences | Specific preferences for how data is displayed in the explanation design, which includes data structures (e.g., free-text, data tables, lists) and graphical representations (e.g., images, plots/charts, diagrams) used to display information. This includes participant preferences for mock-up options (e.g., tornado vs. force plot) and alternative suggestions. Applicable to participant suggestions for new or alternative displays. Generally not applicable to information content preferences or vocabulary/phrasing preferences, use "explanation design--what" and “vocabulary preferences” codes instead. |
| 4.3.2 Vocabulary preferences | Specific preferences for the vocabulary used in the explanation design. Includes how test content is worded (e.g., phrasing used to describe predictors and contributions), expression of numerical information (e.g., risk in probability vs. odds, displaying probability as decimal or percentage), and domain-specific terms/abbreviations that should be used. Often applicable when participants express confusion/difficulties when trying to interpret text/numerical information. |
| 5. Explanation design--what | Generally determined by the answers to the who and why of the context of use, and refers to the content that needs to be included in an explanation. Content of an explanation typically refers to the type of explanation being provided and any information supporting the interpretation of that explanation. This main category code is meant for organizational purposes only and should not be applied. |
| 5.1 Supporting information | Any information that is not a part of the explanation but is required to help support the user's interpretation/understanding of the explanation. This may include things such as source data used in the model or explanation algorithm, supplemental data, and training materials. This parent code should only be applied when a participant comment falls within this parent category but none of its children codes can be applied appropriately. |
| 5.1.1 Interpretation information | Needs for training information on how to interpret explanation information. This includes remarks/questions that indicate participant confusion and/or lack of understanding based on the system design (e.g., trouble interpreting predictors). Not applicable to momentary confusion (i.e., if participant voices question but quickly figures it out themselves). Not applicable to suggestions for data to include in interface to support explanation interpretation (use other “source & supplemental data” code instead). Not applicable to preferences/opinions on system design.  Examples:  -confusion on how to interpret predictor descriptions (e.g., making sense of discretized ranges or feature descriptions)  -confusion on how to interpret predictor contributions and their relation to the baseline and model predictions  Examples where “interpretation information” and “source & supplemental data” (5.1.2) both apply:  -If it was more clear how to interpret xxx information, the xxx information would help me better understand the prediction and/or explanation  -XXX information seems like it might be useful in understanding the prediction and/or explanation, but I find it confusing to interpret  -If the system could include xxx information expressed in yyy manner, it would really help me interpret/understand the prediction/explanation |
| 5.1.2 Source & supplemental data | Preferences/suggestions for including information about the prediction model (e.g., performance statistics, certainty measures, development processes), source data used by the model or explanation algorithm (e.g., raw data used to derive predictors, (un)discretised predictor values, contribution values), or any other supplemental data required to support interpretation of the prediction or explanation (e.g., interventions, care context). Not applicable to suggestions for improvements to the model.  Examples:  -direct/indirect comments on utility of information in explanation plot, predictor table, raw data plots, etc. (e.g., participant uses raw data plot or predictor table to investigate a predictor in explanation plot)  -requests for information on model (e.g., confidence intervals, performance information, feature engineering/selection, etc.)  -requests for information not used by model such as care interventions performed, staffing/triaging/bed assignment procedures that may have affected care, additional patient data needed to interpret prediction, etc.  -comments on utility of diagnosis, demographic & utilization tables  Examples where “interpretation information” (5.1.1) and “source & supplemental data” both apply:  -If it was more clear how to interpret xxx information, the xxx information would help me better understand the prediction and/or explanation  -XXX information seems like it might be useful in understanding the prediction and/or explanation, but I find it confusing to interpret  -If the system could include xxx information expressed in yyy manner, it would really help me interpret/understand the prediction/explanation |
| 5.2 Type of explanation | One part of explanation content refers to the type of explanation that is required, such as whether the explanation is one of processes or behavior and whether it is targeted at the local or global level. Type of explanation can generally be determined by the type of questions the user is asking or the reasoning processes the user is trying to use. This parent code should only be applied when a participant comment falls within this parent category but none of its children codes can be applied appropriately. |
| 5.2.1. Intelligibility query | Specific intelligibility queries (i.e., “inputs”, “outputs”, “certainty”, “why not”/”how to”, “why”, “what if”, “when”) about the system. Includes comments indicating desire to know what data/predictors/inputs are used, what predictions/outputs can be produced, how (un)certain the model is in its predictions, why inputs produce certain outputs or how to get specific outputs, how changing inputs influences outputs, etc. Coding for intelligibility queries in the form of a question should generally not include answers to the question. Often applicable when "seeking information on model development process" code is used. Generally, not applicable to remarks regarding specific design elements.  Examples:  -questions on data/inputs being used by the model  -comments/questions on predictions, including certainty of predictions, how/why predictions are produced, how changes in inputs might influence predictions. |
| 5.2.2 Level & target preferences | Preferences for explanation level (local/global) and target (behavior/processes). Includes comments/questions directly/indirectly expressing an interest in knowing model internals (e.g., weights, mathematical relationships, handling correlated predictors), general trends learned (e.g., how/why the model makes predictions for patient population; general risk factors), and how/why the model makes predictions for individual patients (e.g., patient-specific risk factors). |
| 6. Perceptions of the system | Perceptions of the overall system application. This includes perceptions on the barriers and facilitators to system adoption. For risk prediction models, adoption is closely tied to the utility, credibility, and usability of a model or system. This parent category code should only be applied when a participant comment falls within this parent category but none of its children codes can be applied appropriately. |
| 6.1 Perceptions of system credibility | The credibility, or "believability", of the system. Includes comments on any aspect of the system that may influence the participant’s confidence in prediction accuracy (e.g., high performance may increase confidence, predictors that are outliers/bad data points may decrease it). Not applicable to remarks about the credibility of existing systems, use "prior experiences" code instead. Often applicable with "verification" category codes.  Examples:  -willingness to use/trial system based on performance (e.g., AUC)  -scepticism about model predictions based on identified data errors/biases, missing info, etc.  -comparing model performance/content with domain knowledge or to known model |
| 6.2 Perceptions of system usability | The usability, or ease of use and learnability, of the system (i.e., can the intended goal be accomplished using the system or will users have difficulty?). Includes comments about aspects of the system that facilitate or impede use (e.g., design elements that make information processing easier/harder) and preferences between mock-ups (e.g., saying one mock-up was easier to use/understand than another). Not applicable to remarks about the usability of existing systems, use "prior experiences" code instead. Often applied with “explanation design” codes.  Examples:  -about mock-ups that are easier/harder to use than others  -design elements that exacerbate/alleviate confusion, cognitive effort, time requirements  -design elements that facilitate information synthesis or interpretation |
| 6.3 Perceptions of system utility | The utility, or usefulness, of the system (i.e., is the intended use of the system useful to pursue? will users use it?). Includes suggestions for possible users of the system and comments on the value of system information (e.g., information provided is perceived as informative). Not applicable to remarks about the utility of existing systems, use "prior experiences" code instead. Often applicable with "learning" & "improvement" codes.  Examples:  -suggesting possible applications of the current model/system  -(dis)interest in continued development of system  -(dis)interest in information provided by system (e.g., “it’s not telling me anything new”, “this information could support xxx decision or help me determine xxx faster”) |
