# Supplementary material for: A qualitative research framework for the design of user-centered displays of explanations for machine learning model predictions in healthcare
Source: BMC Med Inform Decis Mak. 2020 Oct 8;20:257. doi: 10.1186/s12911-020-01276-x (PMC7545557; doi:10.1186/s12911-020-01276-x)
Supplement: Supplementary file 4 — Additional file 4. Perceptions of participants on context of use with supporting quotes. Table of findings with supporting quotes for each target question under the context of use portion of the proposed framework. All findings were derived from the analysis of the focus group session transcripts. [file 12911_2020_1276_MOESM4_ESM.docx]

**Perceptions of participants on context of use with supporting quotes**

|  | **Topic** | **Findings** |
| --- | --- | --- |
| **Who** | User Cognition | *Large variation in knowledge of predictive modeling*  “Are we enabling the computer take over?”  “Seems like there’s a lot of collinearity there. And if those are two of the main contributors, I’m wondering whether it’s overestimating”  *Negative experiences with prior systems due to insufficient training, irrelevant information, and inappropriate disruptions in workflow*  “They trigger a sepsis screen every time I do vitals or every 2 hours and you call the doctors and they have to come down and see them and they’re getting really irritated. They don’t want to have to do that, they don’t have time.”  “Most people didn’t know how to use the Rothman index.”  *Clinical role affects how providers anticipate using model information*  “From the attending perspective, when you walk in in the morning who do you need to see first? Who maybe is higher risk than people are appreciating? Or is changed based on data that’s emerged in the last few hours?” |
| **When/where** | Cognitive and time resources | *Providers have limited available time to process information*  “I don’t know if—working on the floor—I get an alert that I have time to go and look through all of this data to try and figure out where the risk is coming from.”  *Providers have limited available cognitive capacity and attention to process information*  “If you load it with a lot of numbers that will probably be not helpful…Because it dilutes your attention.”  “Trying to think about what that actually means…one, you could be wrong, or if it’s 3:00 in the morning…some of the mental gymnastics you’d have to do.” |
|  | Social and organizational influences | *Workflow and social factors determine how providers anticipate using model information*  “Typically, I round on new patients and then I round on any ECMO patients…and then with some filler between, I break up and it’s somewhat random where I start”  “I think it might help with the doctors…If there’s another number—something saying ‘here, look at this, this is actually showing that there’s something going on.’….Because I get push back all the time.” |
| **Why** | Verification | *Providers desired comparisons to existing models and information on model development processes to help validate model*  “And is it better than PRISM or is it the same?”  “We’re assuming that you didn’t just select for the sickest patients in this cohort.”  “Does this weed out their error of charting, things like this?”  *Providers validated model information by comparing to domain knowledge*  “I mean arterial pressure of 250 seems physiologically impossible.”  “The leading variables are patient is having respiratory issues and has kidney injury…from a face validity standpoint—yes, that sounds like a patient with a higher risk of dying.” |
|  | Improvement | *Providers were interested in improving the performance and utility of the model*  “It’d be nice to look at morbidity as well and other things.”  “One of the critical things that you might consider finding a way to incorporate into the score is medications.”  “I do think, from a model validity standpoint, changing this to include maybe abnormal blood pressure for age, does add a lot.” |
|  | Learning | *Providers wanted to use the model to gain insights about patient conditions*  “Does this model offer new information that I didn’t already have? Like ‘this patient was at high risk for mortality and I didn’t otherwise recognize that.’”  “Just telling you what you should know, and what you would appreciate if you clicked into the chart and dove into the information, but at least this is synthesizing that for you.”  *Providers sought actionable information from the model*  “Can I do anything to mitigate that risk of mortality based on what I know?”  “I don’t think there’s a lot I can do about most of that stuff…” |
