# Supplementary material for: A qualitative research framework for the design of user-centered displays of explanations for machine learning model predictions in healthcare
Source: BMC Med Inform Decis Mak. 2020 Oct 8;20:257. doi: 10.1186/s12911-020-01276-x (PMC7545557; doi:10.1186/s12911-020-01276-x)
Supplement: Supplementary file 5 — Additional file 5. Participant preferences on explanation design with supporting quotes. Table of findings with supporting quotes for each target question under the explanation design portion of the proposed framework. All findings were derived from the analysis of the focus group session transcripts. [file 12911_2020_1276_MOESM5_ESM.docx]

**Explanation design preferences with supporting quotes**

|  | **Topic** | **Findings** |
| --- | --- | --- |
| **What** | Type, target, and level of explanation | *Providers sought “input”, “output”, “certainty”, “why not”, and “what if” explanations*  “Do I get a confidence interval somewhere?”  “The first question I get when I talk to the doctor is ‘well, why did that happen?’”  *Some desire for explanations of model processes and at the global-level*  “What’s the weight of the data that is available from the moment they did transfer them to the ICU and how does that carry into this predictive model?”  “Is it possible to see what the machine learned about the relationship between age and raw numbers of vital signs?” |
|  | Supporting information | *Raw feature values, raw time-series data, and contextual information aid interpretation*  “Can I see the data for the blood pressure? Like a 57-point deviation in mean arterial pressure is quite substantial.”  “Everything bad that’s happening with this patient seems to be contributed by the Coma Score…Now I see a diagnosis code of a brain tumor I’d give it much more weight.”  *Providers wanted to see baseline risk and trends of risk prediction over time*  “This doesn’t show like ‘okay, the in-hospital mortality odds, 3 hrs ago was 1.6 and now it's actually coming down?”  “I also think that an odds of death of 12% is still concerning—this child is 10 times more likely to die than the average child in our ICU.”  *Providers stressed importance of proper training on explanation interpretation*  “One risk, I think, with this type of data presentation, is I are going to over-interpret the results…this is just showing you how the model worked, it doesn’t necessarily mean the model is saying you should act on these specific [factors]” |
| **How** | Unit of explanation and organization | *Providers preferred feature groupings for the initial explanation display*  “I really appreciate the groupings on this graph…I said well there’s only an 8% risk of death but it’s being driven largely by this neuro bucket, so what’s going on there?”  *Providers had mixed preferences on organization of predictors*  “I like 2-2…I mean because that’s how my brain thinks—I mean I break things down by that a lot of times in my head—physical assessments, labs, that sort of thing…”  “Being able to see these are the top 5 increase mortality, these are the top 5 lower mortality...as opposed to the 2-2, where I can just see sort of graph fatigue” |
|  | Dimensionality | *Providers wanted interactive linkage of data across plots and tables*  “I also like that clicking on the graph directs you to the associated lab/physical assessment in the data table.”  “Is it possible that when you click on lactate results it could both bring up the raw data graph as well as the little components of the lactate table?  *Interactive control over unit of explanation supported different information needs*  “With the hover capability, if people wanted more, they could have that…I have a basic that everybody can get the basic data, but if you want to dig deeper it’s there.” |
|  | Information representation | *Providers preferred risk to be presented as probabilities expressed as percentages*  “I actually took out a calculator and based on the odds, I calculated the patient’s percent probability of death.”  “For me, percent risk of mortality is going to be easier to interpret than the odds.”  *Providers preferred less statistical terminology for describing trend-based features*  “It would be nice if I could just say, ‘pulse ox is decreasing, creatinine is increasing’”  *Visual cues play an important role in interpretation and design preferences*  “It’s because of the scale of the bars. The tighter bars just don’t grab my attention.”  “I think with mock-up 1-3, my first initial thought is to look—you read left to right. So I would think the SpO monitor results would be the highest contributor.”  *Provider had mixed preferences on explanation display format*  “I think 1-3 actually gives you the most visually, because you can see every element that’s playing into that...I would suspect that there are other people who are going to look at 1-1 or 1-2 and it’s going to be very obvious to them what they’re looking at.”  “In my head, I’m thinking what about a pie chart?” |
